# Adverse Health Events in Chronic Myeloid Leukaemia Patients Treated With Tyrosine Kinase Inhibitors 2009–2019: A Real‐World Study From the UK's Haematological Malignancy Research Network

**DOI:** 10.1002/ijc.70518

**Published:** 2026-04-29

**Authors:** Eleanor Kane, Alexandra Smith, Debra Howell, Catherine Cargo, Kate Rothwell, Simone Green, Russell Patmore, Eve Roman

**Affiliations:** ^1^ Epidemiology and Cancer Statistics Group, Department of Health Sciences University of York York UK; ^2^ Haematological Malignancy Diagnostic Service St James's University Hospital Leeds UK; ^3^ Department of Clinical Haematology Leeds Teaching Hospital NHS Trust Leeds UK; ^4^ Queen's Centre for Oncology and Haematology Castle Hill Hospital Cottingham UK

**Keywords:** cardiovascular events, chronic myeloid leukaemia, morbidity, mortality, tyrosine kinase inhibitor

## Abstract

Outcomes among patients with chronic myeloid leukaemia (CML) improved markedly when tyrosine kinase inhibitors (TKIs) were introduced into routine clinical practice around the turn of the century. Nonetheless, adverse events still occur, survival remains suboptimal and the long‐term health impact of the disease and its treatment is poorly understood. Using data from an established UK population‐based cohort of haematological malignancies, we compared the morbidity of 411 CML patients treated with TKIs 2009–2019 to that of individually age‐ and sex‐matched general population‐based controls (*n* = 4099). Over the course of follow‐up (to March 2021, median 5.3, interquartile range 3.0–8.2 years), patients were more likely than controls to die from cardiovascular or respiratory diseases; Hazard Ratios of 1.9, 95% Confidence Interval, 95% CI = 1.2–2.8 and 2.4, 95% CI 1.2–4.9 respectively. Hospital admissions for cardiovascular or respiratory conditions were similarly elevated; HR = 1.6, 95% CI 1.3–1.9; HR = 2.3, 95% CI 1.6–3.4 respectively. The risk of a cardiovascular admission varied over time; being increased in the first year (HR = 2.4, 95% CI 1.5–3.8), and then again 5 years or more after starting TKI therapy (HR = 2.3, 95% CI 1.5–3.6); no increase was evident in the intervening years (HR = 1.1, 95% CI 0.8–1.7). While not related to excess mortality, admissions for infections and gastrointestinal conditions occurred more frequently among cases than controls; the increased risk remaining largely constant over the course of follow‐up. In the era of TKIs, these real‐world analyses revealed that CML patients are at increased risk of severe cardiovascular events several years after starting treatment, and that admission for infections and gastrointestinal conditions are raised throughout.

## Abstract (highlights)

What's new?

Although tyrosine kinase inhibitors (TKIs) improve chronic myeloid leukemia (CML) outcome, long‐term effects remain unclear, particularly in real‐world settings. Here, the authors examined morbidity and mortality in CML patients treated with TKIs between 2009 and 2019 in the United Kingdom. Relative to the general population, TKI‐treated CML patients experienced increased risk of mortality and hospitalization from cardiovascular and respiratory causes. Cardiovascular disease risk peaked especially around six years after treatment initiation. Hospital admissions for infections and gastrointestinal conditions were also consistently elevated. The findings reveal persistent health risks in TKI‐treated CML patients, underscoring the need for long‐term monitoring.

## Graphical abstract

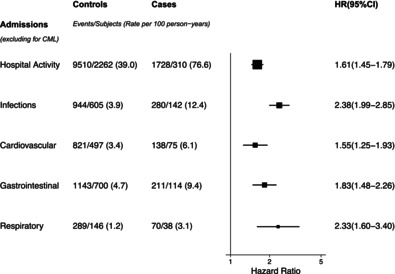

**Abbreviations**
CI: confidence interval
CML: chronic myeloid leukaemia
FG: fibroblast growth factor
HES: Hospital Episode Statistics
HMDS: Haematological Malignancy Diagnostic Service
HMRN: Haematological Malignancy Research Network
HR: hazard ratio
ICD10: International Statistical Classification of Diseases and Related Health Problems 10th Revision
IQR: interquartile range
NHS: National Health Service
OPCS4: OPCS Classification of Interventions and Procedures Version 4
PDGF: platelet‐derived growth factor
TKI: tyrosine kinase inhibitors
VEGF: vascular endothelial growth factors

## 1 Introduction

Introduced into routine clinical practice over 20 years ago, tyrosine kinase inhibitors (TKIs) transformed the prognosis of chronic myeloid leukaemia (CML), changing it from a disease that was often rapidly fatal to one that could be controlled by daily oral therapy [1, 2, 3, 4, 5]. Several next generation TKIs have been developed since the original drug (imatinib) was licensed, and for most patients, TKIs are generally well‐tolerated and severe adverse events are rare [6, 7, 8, 9, 10, 11]. Nonetheless, side effects that result in other symptoms (e.g., muscle cramps, fatigue and nausea) are comparatively common, and health‐related quality of life of CML patients tends to be poorer than that of the general population [12, 13, 14, 15, 16].

On the whole, specific adverse health events have typically been reported within clinical trials where the comparator is often imatinib, and the timeframes are short [8, 9]. More recently, however, longer follow‐up of participants has suggested that TKI treatment may be associated with cardiovascular and arteriothrombotic events [6, 17]. The success of life‐long TKI therapy means that CML prevalence is increasing [18, 19], and there is a clear need for real‐world data to examine health outcomes among all CML patients, regardless of trial participation, age, comorbidities, frailty or time since diagnosis [20]. Thus far, however, only a few studies have compared the health of CML patients to that of the general population [5, 21, 22, 23], resulting in a paucity of knowledge of this topic [24]; data from Sweden revealing increased numbers of cardiovascular events [21] and associations with a wide range of conditions including infections, and respiratory and gastrointestinal disorders [22], and similar observations based on health plan enrolees have been reported in the United States [23], where CML mortality remains higher than that of the general population 5–10 years after diagnosis [5]. With the aim of providing more information about the longer‐term health of CML patients, this richly annotated real‐world UK linkage study compares the morbidity and mortality of CML patients to that of the general population.

## 2 Methods

Data are from the specialist UK population‐based cohort: the Haematological Malignancy Research Network (HMRN: https://hmrn.org/). Initiated in September 2004 with the aim of providing real‐world generalizable data to inform research and clinical practice, details of HMRN's underpinning methods have been published [25, 26]. The cohort's catchment population of around 4 million people is served by 14 hospitals, and all blood cancers and related disorders (~2500 per year) are diagnosed and coded using the latest WHO classifications at a single integrated haematopathology laboratory: The Haematological Malignancy Diagnostic Service (HMDS, https://hmds.info/). Individuals enter the cohort on the day of diagnosis irrespective of age, treatment intent, trial entry, or management within the National Health Service (NHS) or private sector. To facilitate comparisons with the general population, each HMRN patient diagnosed 1 January 2009 to 31 December 2015 was matched to 10 age‐ and sex‐matched unaffected individuals from the same catchment population; the resulting general population cohort containing 181,270 individuals [26]. All patients and corresponding controls are ‘flagged’ via their unique NHS number and followed up for death by NHS England (www.nhsdigital.nhs.uk), and routinely linked to national Hospital Episode Statistics (HES).

This report includes 411 patients aged 18 years or over, diagnosed with CML between 1st January 2009 and 31st August 2019 and treated with TKIs (cases), and 4099 age‐ and sex‐matched individuals from the population cohort (controls) who were alive at the start of their case's TKI treatment. All patients with CML were included regardless of disease phase. To cover the full diagnostic time‐period, controls were assembled in two ways: the 265 CML patients diagnosed 1st January 2009 to 31st December 2015 already had individually matched controls (*n* = 2560) in the general population cohort [26], and the 146 CML patients diagnosed after 2015 had 1460 age‐ and sex‐matched controls randomly selected from unused members of the comparison cohort who were alive when the case was diagnosed. Follow‐up was from the date TKI treatment was initiated (the same date was used for matched controls), and continued until the earliest of death, emigration, or 31 March 2021 (11 controls died before their case started TKI treatment and were excluded from the analysis).

Admissions (day cases and inpatient stays) recorded in HES were analysed for 410 cases and 4088 controls; one case and 11 controls were omitted because they were hospitalized for the entire follow‐up period. Reasons for hospital admission were considered using two separate but complimentary approaches; one based on the primary diagnosis at admission (ICD10, International Statistical Classification of Diseases and Related Health Problems 10th Revision [27]), the other on the primary surgery conducted earliest in each admission (OPCS4, OPCS Classification of Interventions and Procedures Version 4 [28]); admissions for leukaemia were excluded. Cardiovascular, respiratory, or gastrointestinal conditions as well as infections were identified using the ICD10 codes listed in Table S1; and surgical procedures using the OPCS4 codes in Table S2.

To examine CML mortality, particularly the probability of surviving CML in the absence of other causes of death, net survival was calculated relative to national mortality rates [29] using the Pohar Perme method [30]; this relative survival technique was also applied to the controls to estimate their survival standardized to the same age‐ and sex‐national population. Further comparisons of mortality were between cases and controls using Cox regression, where time‐varying hazard ratios were estimated by fitting a continuous smooth regression spline with three degrees of freedom. Underlying causes of death (the disease or injury judged by the certifying clinician to have initiated the train of morbid events leading directly to death) were also examined using Cox regression. Hospital admissions were considered as recurrent events in the analyses and were modelled using conditional frailty regression, where the timescale was days between admissions (or for the first admission, days since starting TKI treatment); baseline hazards were stratified by event number; and heterogeneity in the individuals' event rates was accounted for through a frailty term [31, 32, 33]. Since the gap time scale was used, hazard ratios (HR) and 95% confidence intervals (95% CI) relate to the relative intensity of event occurrence. Time‐varying effects in recurrent events were investigated using semi‐parametric shared frailty models where the time‐varying effect was modelled using regression splines [34]. All analyses were conducted in R using the packages survival, relsurv version 2.2‐9 [30] and frailtypack version 3.7.0 [35].

## 3 Results

Of the 411 CML patients who were treated with TKIs, 55% were male and the median age at diagnosis was 59.3 years (Inter‐Quartile range, 47.0–71.8); demographics were similar for the individually matched controls (Table 1). Median follow‐up was 5.3 years (IQR 3.0–8.2) among cases and around 6.0 (IQR 3.5–8.6) among controls. During this period, 90% of patients (86% at baseline) were prescribed imatinib at some point in time; 39% nilotinib, 26% dasatinib, 12% bosutinib and 6% ponatinib. Almost 55% of patients remained on the same TKI throughout, and 45% switched at least once.

**TABLE 1:** Characteristics of chronic myeloid leukaemia (CML) patients who received tyrosine kinase inhibitors (TKIs) and their matched population controls, HMRN 2009–2019.

	Controls	Cases
Total	4099 (100)	411 (100)
Person‐years^a^	24,445.4	2255.9
Median (IQR)	6.0 (3.5–8.6)	5.3 (3.0–8.2)
Sex, male	2253 (55.0)	226 (55.0)
Female	1846 (45.0)	185 (45.0)
Age at start of treatment		
< 50	1266 (30.9)	128 (31.1)
50–59	837 (20.4)	84 (20.4)
60–69	831 (20.3)	83 (20.2)
70–79	684 (16.7)	68 (16.5)
80+	48 1 (11.7)	48 (11.7)
Age, median (IQR)	59.3 (47.0–71.8)	59.3 (47.0–72.0)
TKIs ever taken		
Imatinib	—	370 (90.0)
Nilotinib	—	160 (38.9)
Dasatinib	—	106 (25.8)
Bosutinib	—	51 (12.4)
Ponatinib	—	25 (6.1)
Deaths	562	114
5‐year overall survival, % (95% CI)	89.4 (88.4–90.5)	77.7 (73.5–82.2)
5‐year net survival, % (95% CI)	100.8 (99.3–102.0)	85.5 (80.2–91.3)
Underlying cause of death^b^		
Chronic myeloid leukaemia	—	37
Leukaemia (not‐CML)	3	—
*Total deaths, excluding leukaemia*	559 (100%)	77 (100%)
Cardiovascular	147 (26.3%)	25 (32.5%)
Cancer	144 (25.8%)	18 (23.4%)
Infection	43 (7.7%)	5 (6.5%)
Respiratory	41 (7.3%)	9 (11.7%)
Other causes	184 (32.9%)	20 (26.0%)

^a^ Follow‐up from date case started TKI to death, 10 years after starting TKI or 31 March 2021.

^b^ Underlying cause recorded on the death certificate = the disease or injury that initiated the train of morbid events leading directly to death (as judged by the certifying clinician).

**FIGURE 1:**
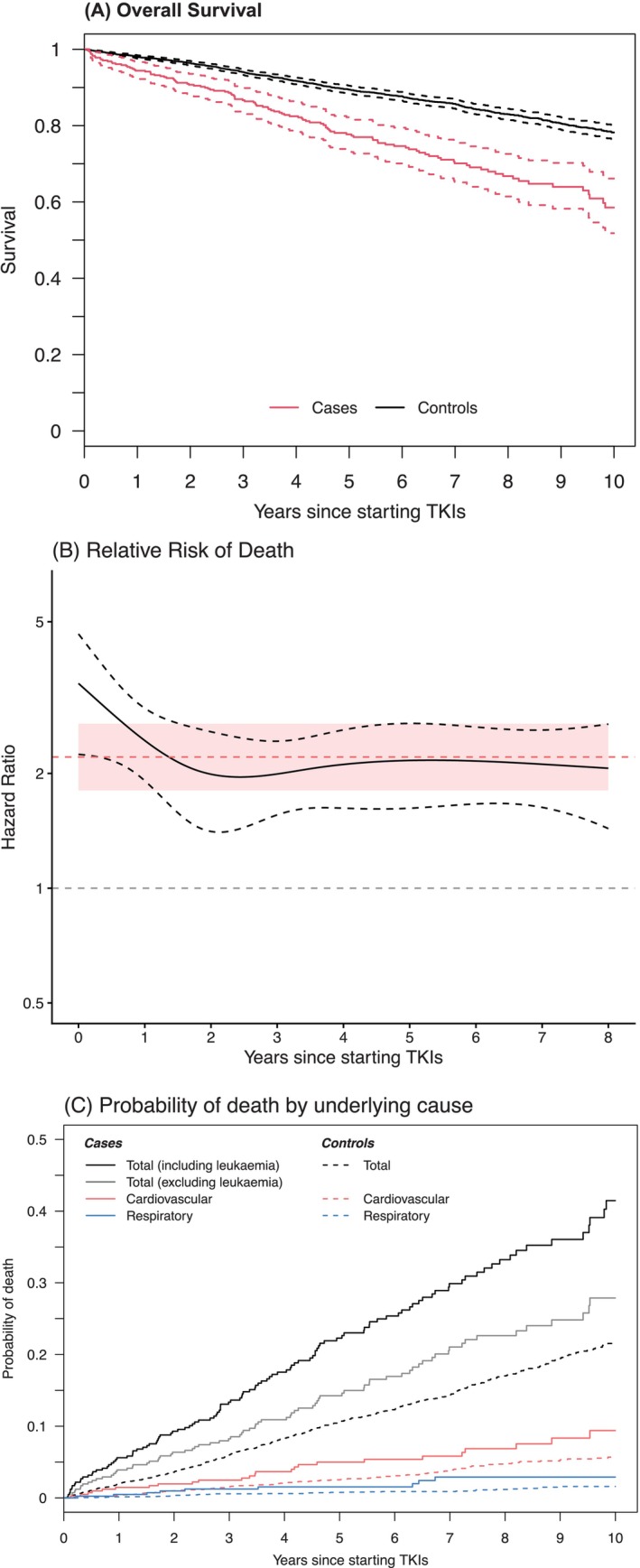
Overall survival (A), relative risk of death (B) and probability of death by underlying cause (C) among chronic myeloid leukaemia patients treated with TKIs (*N* = 411) and their matched controls (*N* = 4099), HMRN 2009–2019, followed for 10 years or to March 2021. (B) Red lines (shading) are the overall estimate (95% confidence interval) with case–control status as a time‐fixed effect, and a reference line at 1.

During follow‐up, 114 (28%) patients and 562 (14%) controls died; yielding 5‐year overall and net survivals of 77.7% (95% Confidence Interval 73.5–82.2) and 85.5% (95% CI 80.2–91.3) respectively for patients. These survival statistics were inferior to those observed among controls‐ 89.4% (95% CI 88.4–90.5) and 100.8% (95% CI 99.3–102.0) respectively. The average hazard ratio (HR) comparing cases to controls was 2.21 (95% CI 1.81–2.70); over the course of follow‐up, survival remained poorer than among controls (Figure 1A), the HR being higher at the start of follow‐up but still elevated several years after starting TKI treatment (Figure 1B). Examination of the clinical records of the 37 patients where CML was recorded as the underlying cause on the death certificate confirmed that 13 died during a blast/accelerated phase, four developed graft versus host disease and five were treatment non‐compliant. CML was well‐controlled in two patients, but in both, the immediate cause of death (severe bleeding, one gastric and one cerebral) was recorded as being due to CML on the death certificate. Clinical monitoring/compliance data for the remaining 13 patients, all of whom were elderly/frail, were not available in the months leading up to death. The underlying cause of the majority of deaths among patients (77/114) was attributed to conditions other than CML (Table 1, Figure 1C); although CML was recorded as an intermediary cause in just over half (*n* = 42). The hazard ratios (HR) for death from cardiovascular and respiratory diseases were notably high at 1.86 (95% CI 1.22–2.84) and 2.38 (95% CI 1.16–4.90) respectively, whereas deaths due to cancers other than leukaemia, infection and other causes were broadly similar.

**FIGURE 2:**
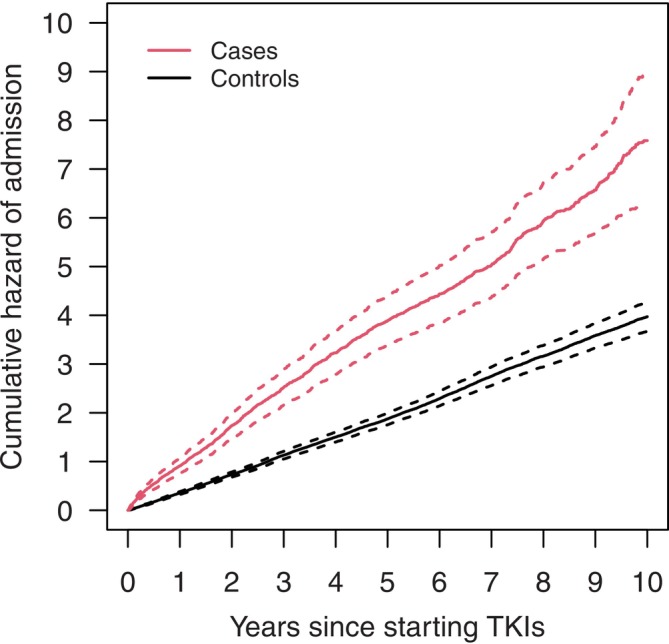
Cumulative hazard of admissions for conditions other than leukaemia among TKI treated patients with chronic myeloid leukaemia (*N* = 410) and their matched controls (*N* = 4088), HMRN diagnoses 2009–2019 followed to March 2021.

**FIGURE 3:**
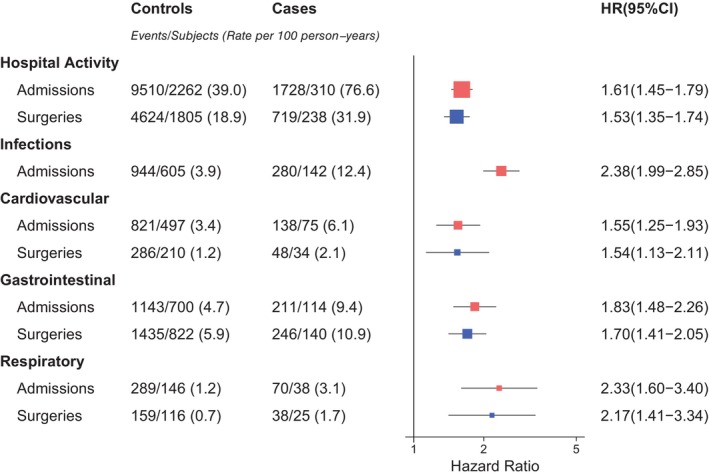
Hazard ratios of admissions for conditions and surgeries among chronic myeloid leukaemia patients (*N* = 410) treated with tyrosine kinase inhibitors compared to their matched controls (*N* = 4088), HMRN 2009–2019 followed to March 2021.

Over the follow‐up period, the cumulative hazard of hospital admission for conditions other than leukaemia (Figure 2) was significantly higher among cases than controls (HR = 1.61, 95% CI 1.45–1.79). The risk of admission involving surgery was also higher (HR = 1.53, 95% CI 1.35–1.74) (Figure 3). Infection was the most common reason for admission among cases, with 280 admissions occurring among 142 (34%) CML patients, compared to 944 among 605 (15%) controls (HR = 2.38, 95% CI 1.99–2.85). Cardiovascular admissions occurred 138 times among 75 cases, compared to 821 times among 497 controls (HR = 1.55, 95% CI 1.25–1.93); although cardiovascular surgery was conducted on fewer occasions and among fewer subjects, the risk estimate was similarly increased (HR = 1.54, 95% CI 1.13–2.11). Gastrointestinal problems and surgical procedures were also relatively common, occurring in over a quarter of cases and around a fifth of controls (HR = 1.83, 95% CI 1.48–2.26; HR = 1.70, 95% CI 1.41–2.05, respectively). With regard to respiratory conditions, there were 70 admissions among 38 cases, and 289 admissions among 146 controls (HR = 2.33, 95% CI 1.60–3.40); respiratory surgery was less common (38 among 25 cases and 159 among 116 controls), yielding an HR of 2.17 (95% CI 1.41–3.34).

**FIGURE 4:**
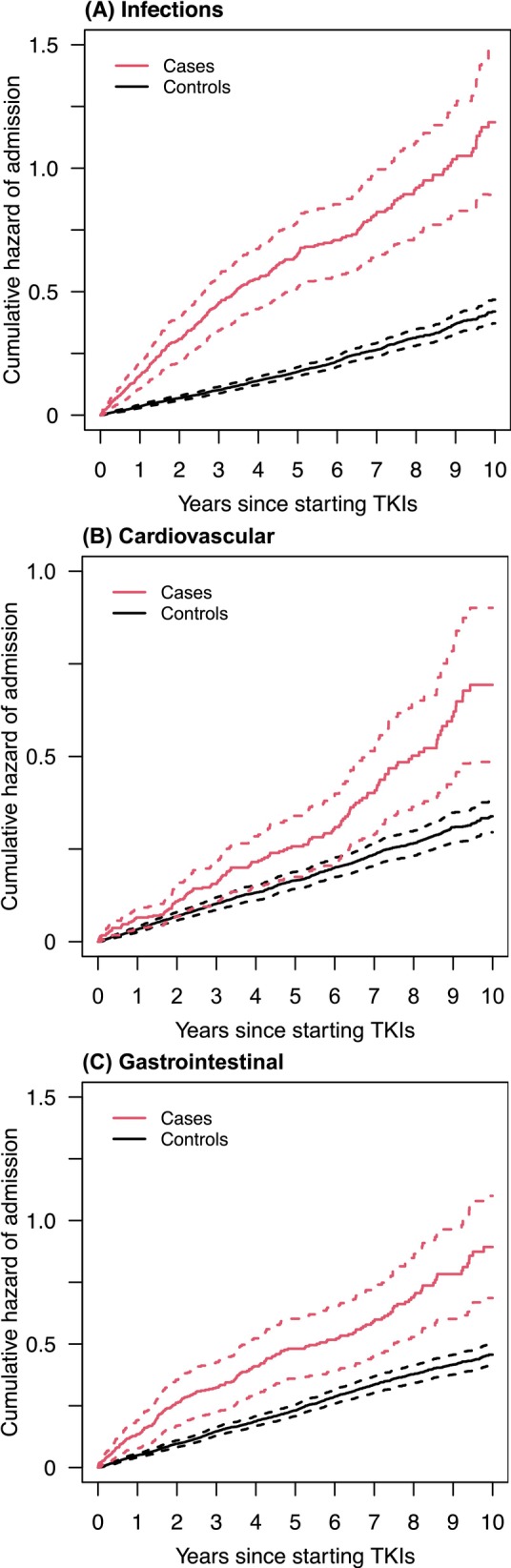
Cumulative hazard of admissions for infections (A); cardiovascular disease (B) and gastrointestinal conditions (C) among chronic myeloid leukaemia patients (*N* = 410) relative to starting treatment with tyrosine kinase inhibitors compared to their matched controls (*N* = 4088) (pseudo‐treatment start date for controls), HMRN 2009–2019 followed to March 2021.

Figure 4 plots the cumulative hazard for infections, cardiovascular disease and gastrointestinal conditions; respiratory diseases are not shown due to the low rates of admission. For infections and gastrointestinal conditions, the slope of the cumulative hazard was consistently higher among cases than controls (Figure 4A,C respectively). For cardiovascular disease, however, there is a marked divergence in the gradient around 6 years after the start of treatment (Figure 4B). Modelling the relative differences in recurrent admissions confirmed that the time‐varying HRs were consistent with these patterns; remaining largely constant for infections and gastrointestinal conditions, but increasing after 5–6 years for cardiovascular diseases (Figure S1). The variation in cardiovascular disease risk over time was examined further in a time‐to‐first event analysis, being elevated in the first year (HR = 2.43, 95% CI 1.54–3.82), and again 5 years or more after starting TKIs (HR = 2.30, 95% CI 1.49–3.55), with little difference between cases and controls in the intervening years (HR = 1.14, 95% CI 0.77–1.69).

Myocardial ischaemia (HR = 2.29, 95% CI 1.59–3.30 based on 47 admissions among 29 cases vs. 180 among 126 controls), stroke (HR = 1.77, 95% CI 1.06–2.97, 17 among 16 cases vs. 98 among 89 controls) and heart failure (HR = 1.99, 95% CI 1.13–3.48, 18 among 16 cases vs. 85 among 62 controls) were among the most common reasons for cardiovascular admission. Cardiovascular surgery, which included heart surgery and procedures to revascularize lower limbs, was also conducted more often among cases (heart surgery: HR = 1.85, 95% CI 1.14–3.03, 19 among 16 cases vs. 106 among 95 controls and lower limb‐saving surgery: HR = 2.38, 95% CI 1.11–5.11, 9 among 4 cases vs. 33 among 26 controls). Apart from cardiovascular disease, other serious conditions that were elevated included renal failure (HR = 3.13, 95% CI 1.90–5.16, 24 among 17 cases vs. 60 among 51 controls), and pleural effusion, either as the admitting condition or as an aspiration procedure (HR = 8.41, 95% CI 4.12–17.2, 17 among 14 cases vs. 18 among 15 controls; HR = 4.74, 95% CI 2.48–9.04, 22 among 15 cases vs. 37 among 28 controls, respectively).

## 4 Discussion

Tracking all hospital admissions, surgeries and deaths, our population‐based data reflect the real‐world situation for CML patients, showing the reality of their health patterns. Whilst providing reassurance overall, with a 5‐year net survival of 85.5% (95% CI 80.2–91.3), mortality nonetheless remained higher than the general population several years after starting TKI treatment. This increase in mortality was due, at least in part, to higher risks of cardiovascular and respiratory deaths. When examining morbidity, events involving the cardiovascular system (admissions for myocardial ischaemia, stroke and lower limb operations) were amongst the most severe, with risks increasing markedly around 5–6 years after TKI treatment was first initiated. Furthermore, whilst not related to excess mortality, hospitalization for infections and gastrointestinal conditions were consistently significantly increased from the start of treatment onwards. Previous evidence has tended to focus on events during specific TKI treatments [17, 21, 23], and on the risk of an event's first occurrence [17, 22, 23]; whereas the findings presented here relate to the longer term morbidity burden, from the start of CML treatment until 10 years later.

Following standard protocols, most UK patients are initially treated with imatinib (Glivec/Gleevec; Novartis), with only a small proportion solely treated with later generation first‐line alternatives, commonly nilotinib (Tasigna; Novartis) and dasatinib (Sprycel; Bristol‐Myers Squibb), which may also be offered when initial imatinib treatment fails, or at the development of side effects [36]. We found that 10 of the 17 admissions for pleural effusion were among patients who were taking dasatinib at the time, and eight of the nine limb procedures involved patients who had been treated with nilotinib for a number of years. Others have suggested that cardiovascular conditions may occur more often among those treated with later generation TKIs, but such events have still been recorded amongst those on imatinib [6, 7, 17, 21, 22, 23, 37, 38]. Nearly half of patients in our cohort switched TKI at least once during follow‐up, and events were seen amongst patients whether they were treated with one, or with two or more TKIs; amongst the 75 patients with a cardiovascular admission for instance, 45 only received one TKI before their first admission (34 with imatinib, 6 dasatinib and 5 nilotinib), the remaining 30 received imatinib and at least one other TKI (dasatinib and/or nilotinib). As such, attributing adverse events to specific TKIs is challenging, and other aspects such as duration of treatment, as well as other potentially confounding factors may need to be considered.

Designed to control ABL kinase, TKIs for CML have been found to inhibit a spectrum of other kinases, including receptors for vascular endothelial growth factors (VEGF), TIE2, platelet‐derived growth factor (PDGF), stem cell factor (c‐KIT) and fibroblast growth factor (FGF) [39, 40, 41]. With kinase enzymes being critical to cardiac, vascular and metabolic homeostasis, inhibitions of these off‐target kinases are thought to act via multifaceted mechanisms to cause cardiovascular problems, through dysfunction of cardiomyocytes, the endothelium and the signalling pathways of cardiac function [42, 43, 44, 45, 46]. Although such dysfunctions can be reversed, it is possible that continued TKI exposure could result in the manifestation of symptomatic diseases, such as those we have observed. Indeed, since cardiovascular disease is increasingly being recognized as a potential consequence of CML treatment, risk management for such conditions has been incorporated into recent guidelines [2, 6, 36, 47]; the data here providing a base against which the future impact of their implementation into routine CML care can be compared [48].

In summary, our real‐world longitudinal analysis adds to the accumulating body of evidence on the long‐term mortality and morbidity of CML patients. Major strengths of our patient cohort include the large well‐defined catchment that is socio‐demographically representative of the UK as a whole. Served by a centralized haematopathology laboratory, all newly diagnosed haematological malignancy patients within the area are routinely linked to national healthcare datasets, as are the members of our sex‐ and age‐matched general population comparator cohort. Our population‐based CML cohort includes all patients treated with TKIs, the majority of whom were chronic phase. Furthermore, at 100.8% (95% CI 99.3–102.0), the 5‐year net survival of our controls further evidences the fact that our comparator cohort is representative of the country as a whole [30]. With respect to weaknesses, the administrative data used in this report are subject to the same inconsistencies in recording and coding that affect all linkage studies of this type. Information contained within HES, for example, is collected for clinical and payment purposes, rather than for research. As such, it is possible that the presence of CML may have affected which condition was recorded as the primary reason for an admission. However, since the hazard ratios based on major surgical procedures were of similar magnitude, systematic biased recording seems unlikely. Infections were those recorded as the main reason for admission, and did not include those which occurred during an admission, were secondary to another condition, nor those which did not require hospitalization; as such, our findings do not relate to an infection rate per se. Reliable information on comorbidities was lacking; and although perhaps a crude surrogate, stratifying risks by age at diagnosis (age < 60/≥ 60 years) showed little discernible differences (data not shown). With respect to mortality, our analysis examined the underlying cause of death (cause identified as the one that initiated the train of events leading directly to death), which is used globally in routinely compiled mortality statistics and public health research [49]. In some patients, the recording of CML may have taken precedence over other causes. However, through additional clinical data in our patient register, we were able to confirm that CML was the cause of 22 of the 37 deaths (60%) where it was listed as the underlying cause, but insufficient information was available on the remaining 15 patients, who were often in residential care at the time of death. Nonetheless, whilst separating underlying, immediate and intermediate causes presents recognized challenges [50, 51, 52], it remains the case that mortality, along with morbidity, is increased in patients with CML.

Notably, this is the first study to examine the burden of morbidities among CML patients over time; finding that cardiovascular events may occur 6 or more years after treatment begins, while admissions for infections and gastrointestinal conditions occur with relative consistency throughout the follow‐up period. However, with 90% of patients (86% at baseline) being prescribed imatinib at some point in time, and only 55% remaining on the same TKI throughout, the potential aetiological roles of particular TKIs are difficult to disentangle. Nonetheless, our findings provide useful information on the health of those living with CML that could impact future monitoring policies.

## Author Contributions

**Eleanor Kane:** conceptualization, formal analysis, writing – original draft, writing – review and editing. **Alexandra Smith:** conceptualization, data curation, project administration, writing – review and editing, funding acquisition. **Debra Howell:** data curation, writing – review and editing, project administration. **Catherine Cargo:** data curation, writing – review and editing. **Kate Rothwell:** data curation, writing – review and editing. **Simone Green:** data curation, writing – review and editing. **Russell Patmore:** data curation, writing – review and editing, conceptualization. **Eve Roman:** conceptualization, funding acquisition, writing – original draft, writing – review and editing.

## Funding

This work was supported by Cancer Research UK and Blood Cancer UK (Grant Number 29685). E.R. and A.S. are supported in part by the National Institute for Health and Care Research (NIHR) Leeds Biomedical Research Centre (BRC) (NIHR203331).

## Disclosure

The views expressed are those of the author(s) and not necessarily those of the NHS, the NIHR or the Department of Health and Social Care.

## Ethics Statement

Haematological Malignancy Research Network has full ethical approval (Leeds West Research Ethics Committee 04/Q1205/69) including Section 251 support under the NHS Act 2006 (NHS Health Research Authority Confidentiality Advisory Group 20/CAG/0149).

## Conflicts of Interest

The authors declare no conflicts of interest.

## Supporting information


**Table S1:** International Statistical Classification of Diseases and Related Health Problems 10th Revision (ICD10) codes used in analyses.
**Table S2:** Office of Population Censuses and Surveys Classification of Interventions and Procedures Version 4 (OPCS4) codes used in analyses.
**Figure S1:** Time‐varying hazard ratios and 95% confidence intervals for recurrent admissions for infections, cardiovascular and gastrointestinal conditions.

## Data Availability

Ethical approvals and data privacy restrictions limit data sharing to collaborative projects. Further information is available from the corresponding author upon request.
