# Supplementary material for: Adverse Health Events in Chronic Myeloid Leukaemia Patients Treated With Tyrosine Kinase Inhibitors 2009–2019: A Real‐World Study From the UK's Haematological Malignancy Research Network
Source: Int J Cancer. 2026 Apr 29;159(6):1411–9. doi: 10.1002/ijc.70518 (PMC13397138; doi:10.1002/ijc.70518)
Supplement: Supplementary file 1 — Table S1: International Statistical Classification of Diseases and Related Health Problems 10th Revision (ICD10) codes used in analyses. Table S2: Office of Population Censuses and Surveys Classification of Interventions and Procedures Version 4 (OPCS4) codes used in analyses. Figure S1: Time‐varying hazard ratios and 95% confidence intervals for recurrent admissions for infections, cardiovascular and gastrointestinal conditions. [file IJC-159-1411-s001.pdf]

Adverse health events in chronic myeloid leukaemia patients treated with tyrosine kinase inhibitors 2009-2019: a real-world study from the UK's Haematological Malignancy Research Network

Eleanor Kane, Alexandra Smith, Debra Howell, Catherine Cargo, Kate Rothwell, Simone Green, Russell Patmore, Eve Roman

Supplementary Table 1: International Statistical Classification of Diseases and Related Health Problems 10th Revision (ICD10) codes used in analyses.......... 2

Supplementary Table 2: Office of Population Censuses and Surveys Classification of Interventions and Procedures Version 4 (OPCS4) codes used in analyses. 3

Supplementary Figure 1: Time-varying hazard ratios and 95% confidence intervals for recurrent admissions for infections, cardiovascular and gastrointestinal conditions.......... 4

Supplementary Table 1: International Statistical Classification of Diseases and Related Health Problems 10th Revision (ICD10) codes used in analyses.

Main Category	ICD10 Codes
Infection	A00-A99,<br>B00-B99,<br>G00-G02, G04.1-G04.2, G06-G07, G53.0-G53.1, G63.0, G73.4,<br>H00.0, H03.0-H03.1, H05.0-H05.1, H06.1, H10.0, H13.0-H13.1, H18.1, H19.0-H19.2, H45.1, H48.0, H58.8,<br>H60.0-H60.3, H62.0-H62.4, H66, H67.1, H70, H73.0-H73.1, H75.0, H94.0,<br>I00-I09, I30.1, I32.0-I32.1, I33.0, I39, I40.0, I41.0-I41.2, I52.0-I52.1,<br>J00-J15, J16.0, J16.8, J17.0-J17.3, J17.8, J18-J22, J31-J32, J34.0, J35.0, J36, J37.1, J40-J42, J85, J86.9,<br>K02, K04.1-K04.2, K04.5-K04.7, K05.0-K05.3, K10.2-K10.3, K11.2-K11.3, K12.2, K23.0-K23.1, K35-K37, K61, K63.0, K65.0, K67, K75.0, K77.0, K81.0, K83.0, K87.1, K93.0-K93.1,<br>L00-L04, L05.0, L08,<br>M00, M46.2-M46.3, M46.5, M60.0, M65.1, M71.0, M86,<br>N10-N12, N30.0, N30.9, N34.0, N39.0, N41, N43.1, N45, N48.2, N49, N61, N70-N73, N75.1, N75.8, N76,<br>O23, O70, O75, O80, O83, O85-O86, O91, O98,<br>U07.1-U07.2
Cardiovascular	I10-I28, I30.0, I30.2, I30.8-I30.9, I31, I32.8, I33.9, I34-I38, I40.1, I40.8-I40.9, I41.8, I42-I51, I52.8, I53-I99,<br>G45-G46
Gastrointestinal	K00-K01, K03, K04.0, K04.3-K04.4, K04.8-K04.9, K05.4-K05.6, K06-K09, K10.0-K10.1, K10.8-K10.9, K11.0-K11.1, K11.4-K11.9, K12.0-K12.1, K12.3, K13-K14, K20-K22, K23.8, K25-K31, K38, K40-K46, K50-K52, K55-K60, K62, K63.1-K63.5, K63.8-K63.9, K64, K65.8-K65.9, K66, K70-K74, K75.1-K75.4, K75.8-K75.9, K76, K77.8, K80, K81.1, K81.8-K81.9, K82, K83.1-K83.5, K83.8-K83.9, K85-K86, K87.0, K90-K92, K93.8
Respiratory	J30, J33, J34.1-J34.3, J34.8, J35.1-J35.3, J35.8-J35.9, J37.0, J38-J39, J43-J84, J86.0, J87-J99
**Subcategory**	**ICD10 Codes**
Myocardial Ischaemia	I20-I25, except I25.2
Other Heart Disease	I30-I52, except I30.1, I32.0-I32.1, I33.0, I39, I40.0, I41.0-I41.2, I52.0-I52.1
Stroke	I60-I67
Pleural Effusion	J90-J91
Renal failure	N17-N19

Supplementary Table 2: Office of Population Censuses and Surveys Classification of Interventions and Procedures Version 4 (OPCS4) codes used in analyses.

Main Category	OPCS4 Codes
Cardiovascular	K01-K62, K64-K78, L01-L90, L93-L99, O01-O05, O15, O20, X09-X11
Gastrointestinal	G01-G21, G23-G38, G40-G80, H01-H37, H40-H47, H50-H55, H56.2-H56.4, H56.8-H56.9, H57-H71, J01-J63, J65-J70, J72-J77, T20-T23, T27-T28, T30-T48, T51, T98
Respiratory	E01-E08, E10-E57, E59, E61-E65, E67, T01-T17
**Subcategory**	
Heart surgery	K25-K31, K34, K36, K38, K40-K47, K59.1-K59.2, K59.6, K60.1, K60.5-K60.7, K61.1, K61.5-K61.7, K72.1, K75
Revascularisation of lower limb	L16, L20.6, L21.6, L50-L52, L54, L58-L60, L63, L65, L66.2, L66.5, L66.7-L66.9, L68, L71.1, L71.8-L71.9, X09
Pleural aspiration	T08, T12.2-T12.4, T12.6
**Excluded Codes**	
Investigations and procedures patients with CML may undergo	A54-A55, A57.4, A57.7, L91-L92, L99.7, W36, X36, X70-X74
Non-Surgical	A83, C87, E85, E87, E89, E91-E95, E97-E98, F43, K63, M47, N35, O12, O16, O30, P26, R03, R15, R19, R20, R23, R24, R36-R38, R40, R42, R43, S12, S58, T90, U01, U04-U37, U40, U50-U54, V55, X28-X35, X37-X40, X43-X44, X47-X52, X56, X58, X60-X62, X65-X68, X82-X87, X89-X93, X95-X96

Supplementary Figure 1: Time-varying hazard ratios and 95% confidence intervals for recurrent admissions for: infections (A); cardiovascular (B); and gastrointestinal conditions (C). Time is relative to starting treatment with tyrosine kinase inhibitors or since the previous admission. Red lines (shading) are the overall estimate (95% confidence interval) with case-control status as a time-fixed effect, and a reference line at 1.

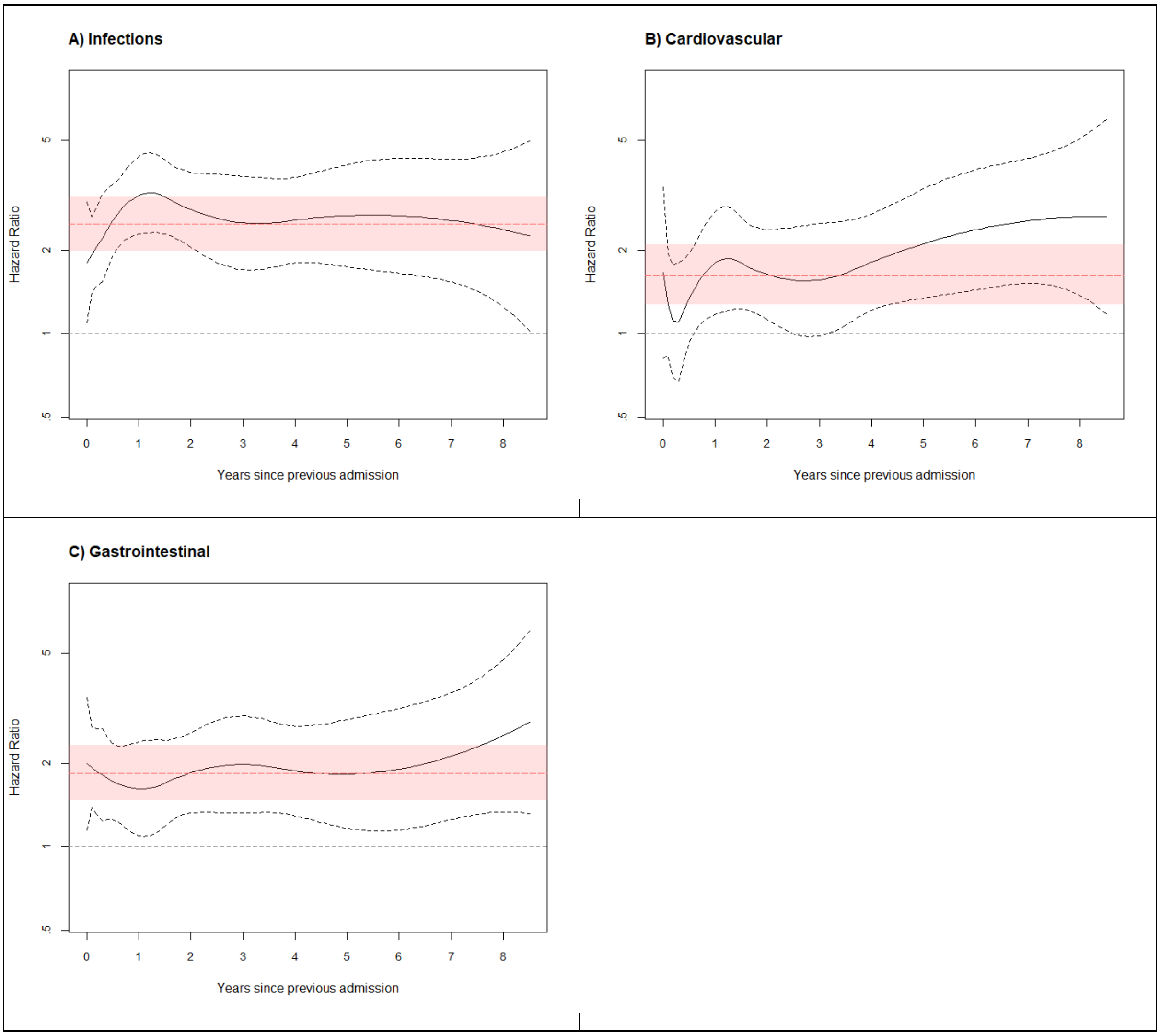

Time-varying effects of case-control status were fitted using semi-parametric shared frailty models with 3 inner knots and order of 3 for quadratic B-splines. The time scale was the gaps between admissions, or for the first event, the time since starting tyrosine kinase inhibitors. Models included stratification for having a previous admission or not; adjustment for the number of previous admissions; and a frailty term to account for heterogeneity in the subject-specific admission rates.
